# Acceptability, Safety, and Efficacy of Oral Administration of Extracts of Black or Red Maca (*Lepidium meyenii*) in Adult Human Subjects: A Randomized, Double-Blind, Placebo-Controlled Study

**DOI:** 10.3390/ph9030049

**Published:** 2016-08-18

**Authors:** Carla Gonzales-Arimborgo, Irma Yupanqui, Elsa Montero, Dulce E. Alarcón-Yaquetto, Alisson Zevallos-Concha, Lidia Caballero, Manuel Gasco, Jianping Zhao, Ikhlas A. Khan, Gustavo F. Gonzales

**Affiliations:** 1 Department of Biological and Physiological Sciences, Faculty of Sciences and Philosophy, Universidad Peruana Cayetano Heredia, Av. Honorio Delgado 430, Lima 31, Peru; carla.gonzales@upch.pe (C.G.-A.); dulce.alarcon@upch.pe (D.E.A.-Y.); alisson.zevallos.c@upch.pe (A.Z.-C.); manuel.gasco@upch.pe (M.G.); 2 Research Circle on Plants with Effects on Health, Lima 15102, Peru; irmaundac@hotmail.com (I.Y.); elsamonterojara@hotmail.com (E.M.); 3 Instituto de Investigaciones de la Altura, Universidad Peruana Cayetano Heredia, Lima, 15102, Peru; 4 Facultad de Enfermeria, Universidad Nacional Daniel Alcides Carrión, Pasco 19001, Peru; 5 Facultad de Nutrición, Universidad Nacional del Altiplano, Puno 21001, Peru; lidia.caballero.g@upch.pe; 6 National Center for Natural Products Research, Research Institute of Pharmaceutical Sciences, University of Mississippi, Oxford, MS 38677, USA; jianping@olemiss.edu (J.Z.); ikhan@olemiss.edu (I.A.K.)

**Keywords:** toxicity, adverse effects, red maca, black maca, *Lepidium meyenii*, placebo, high altitude, human health, nutraceuticals, botanicals

## Abstract

The plant maca, grown at 4000 m altitude in the Peruvian Central Andes, contains hypocotyls that have been used as food and in traditional medicine for centuries. The aim of this research was to provide results on some health effects of oral administration of spray-dried extracts of black or red maca (*Lepidium meyenii*) in adult human subjects living at low (LA) and high altitude (HA). A total of 175 participants were given 3 g of either placebo, black, or red maca extract daily for 12 weeks. Primary outcomes were changes in sexual desire, mood, energy, health-related quality of life score (HRQL), and chronic mountain sickness (CMS) score, or in glycaemia, blood pressure, and hemoglobin levels. Secondary outcomes were acceptability and safety, assessed using the Likert test and side effect self-recording, respectively, and the effect of altitude. At low altitude, 32, 30, and 32 participants started the study receiving placebo, red maca, or black maca, respectively. At high altitudes, 33, 35, and 31 participants started the study receiving placebo, red maca, and black maca, respectively. Consumption of spray-dried extracts of red and black maca resulted in improvement in mood, energy, and health status, and reduced CMS score. Fatty acids and macamides were higher in spray-dried extracts of black maca than in red maca. GABA predominated in spray-dried extracts of red maca. Effects on mood, energy, and CMS score were better with red maca. Black maca and, in smaller proportions, red maca reduced hemoglobin levels only in highlanders with abnormally high hemoglobin levels; neither variety of maca reduced hemoglobin levels in lowlanders. Black maca reduced blood glucose levels. Both varieties produced similar responses in mood, and HRQL score. Maca extracts consumed at LA or HA had good acceptability and did not show serious adverse effects. In conclusion, maca extract consumption relative to the placebo improved quality of life parameters. Differences in the level of improvement between red and black maca are probably due to differences in the composition of these two plant varieties. Both maca extracts were well tolerated and safe.

## 1. Introduction

Maca (*Lepidium meyenii*) is a plant grown at over 4000 m in Peru that belongs to the Brassicaceae family (Cruciferous vegetables). It has been consumed for centuries as food by inhabitants of the Peruvian Central Andes due to the nutritional and medicinal properties of its hypocotyls [1].

Maca is present in nature in different external colors or phenotypes [2]. Several studies showed that different colors of maca produce different biological responses. In fact, experimental studies showed that red maca reverses testosterone-induced prostatic benign hyperplasia [3] and ovariectomized-induced osteoporosis in adults’ rats [4], whereas black maca increases sperm count, reduces glycemia in streptozotocin-induced diabetes mellitus [5], and enhances memory and learning [6] in experimental animal models.

An in vitro study showed that maca decreased angiotensin-converting enzyme (ACE) activity, thereby reducing the availability of angiotensin, a hormone with hypertensive properties [7]. Similarly, in a population traditionally consuming maca, aged 40–70 years, a lower systolic blood pressure but not diastolic blood pressure was observed compared with a non-consuming population [8].

A cross-sectional study showed that maca consumption was associated with high scores on a health-related quality of life (HRQL) questionnaire (SF-20) in men and women aged 40–70 years living at high altitude [8]. This high health status observed in maca consumers was also associated with low levels of cytokine interleukin 6 (IL-6) [8]. These results are in concordance with data showing that consumption of higher amounts of cruciferous vegetables was associated with reduced systemic levels of proinflammatory cytokines as interleukin 6 (IL-6) [9,10] suggesting that cruciferous vegetables are healthy foods.

Chronic mountain sickness (CMS) is a pathology observed only in populations living at high altitude (HA) and represents a lack of adaptation to live at high elevations [11]. In a cross-sectional study at high altitudes, maca consumption was associated with reduced prevalence of CMS in adult men and women, based on a questionnaire of seven signs/symptoms of CMS and elevated hemoglobin levels [12].

In humans, self-perception of sexual desire increased after eight weeks of consumption of gelatinized maca [13] or four weeks after consumption of maca extracts [14]. Blood pressure decreases with maca treatment in menopausal women [15]. However, it is unknown yet if such an effect depends on the different phenotypes of maca.

Since most of the results were based on a cross-sectional design, it is unlikely that we can prove a cause and effect relationship for consuming maca. For this reason, a prospective study is necessary to determine the efficacy on health status. In addition, interest in maca as a food supplement has increased worldwide, and so it is necessary to ensure the acceptability and safety of the products during its consumption.

The present work describes the possible effects of oral administration of spray-dried extracts of black or red maca (*Lepidium meyenii*) in adult human subjects placed in two areas: one located in the Peruvian central Andes, close to where maca is cultivated and where consumption is traditional, and a second population located at the coast where maca consumption is less common. This study assesses as primary outcomes the efficacy of spray-dried extracts of black or red maca on variables associated with health status and as secondary outcomes the acceptability of consumption of black maca or red maca extract as compared with a placebo. In addition, the study aims to describe the self-reported acceptability and side effects after oral administration of spray-dried extracts of black or red maca (*Lepidium meyenii*) in adult human subjects living at low (LA) and high altitude (HA) during 12 weeks in a randomized, double blind, placebo-controlled trial. It was hypothesized that several variables associated with health status would improve more with spray-dried maca extracts than with the placebo. It was also expected that acceptability would be higher at high altitude than at low altitude and that effect size would be different between red and black maca extracts but similar between altitudes.

## 2. Results

### 2.1. Composition of Spray-Dried Maca Extracts

Nuclear magnetic resonance profiles of spray-dried extracts of black and red maca are found in Table 1. Sixteen compounds were identified in both maca extracts. Present in both extracts were the maca metabolites macamides and the main glucosinolate, glucotropaeolin.

Some differences were observed in the composition of spray-dried extracts of black maca and red maca. Fatty acids and macamides were higher in black maca than in red maca. GABA predominated in red maca than in black maca. Other compounds such as phytosterols, uridine, and choline were present in equal amounts in both extracts.

### 2.2. Primary Outcomes: Efficacy of Maca Consumption on Several Variables Associated with Heath Status

#### 2.2.1. Assessment of Sexual Desire

**Figure 1:**
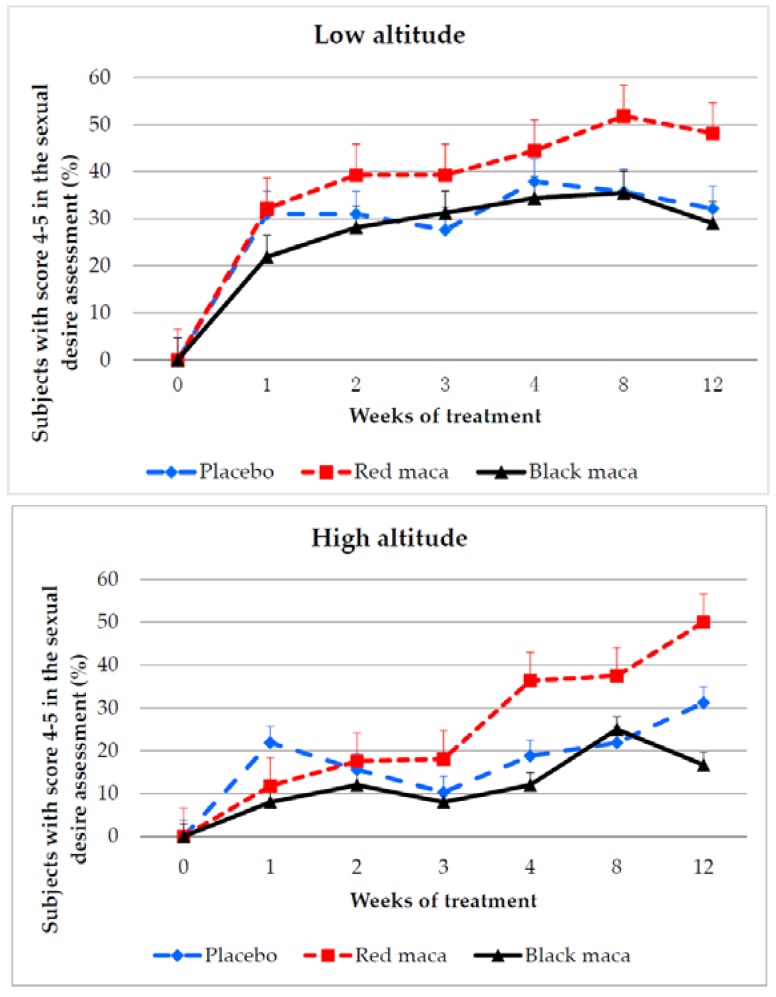
Percentage of subjects perceiving an increase in sexual desire following a 12-week treatment with the placebo or a spray-dried extract of red or black maca at low (**Upper**) and high altitudes (**Bottom**). Bars are standard error. At LA, consumption of spray-dried extract of red maca increases the percentage of subjects with increased sexual desire over time (*p* = 0.0055). *p* > 0.05 between placebo and maca-treated groups. HA: *p* = 0.0027, *p* = 0.0000, and *p* = 0.0019 over time for treatments with placebo, red maca, and black maca, respectively. *p* = 0.03 (chi square = 6.96) at week 12 between treatment with spray-dried extract of red maca and placebo or spray-dried extract of black maca.

At LA, red maca and black maca increased perception of sexual desire over time from week 1 to week 12. Although the percentage of people reporting sexual desire improvement is higher in the group treated with red maca from week 2 to week 12, this difference was not significant with respect to the placebo group (Figure 1 upper). Effect size resulted in a Cohen’s *d* = 0.37 between the placebo and red maca and Cohen’s *d* = 0.44 between red maca and black maca.

At HA, the number of subjects with scores of 4–5 for sexual desire after treatment with the placebo, red maca, or black maca increased over time. Differences between response about sexual desire in subjects treated with red maca and the placebo were observed at week 12 (*p* = 0.03) (Figure 1 bottom). Effect size resulted in a Cohen’s *d* = 0.436 between placebo and red maca and 0.88 between red and black maca.

Around 50% of subjects at both altitudes reported an increase in sexual desire at week 12 of treatment with spray-dried red maca extract.

#### 2.2.2. Assessment of Mood

**Figure 2:**
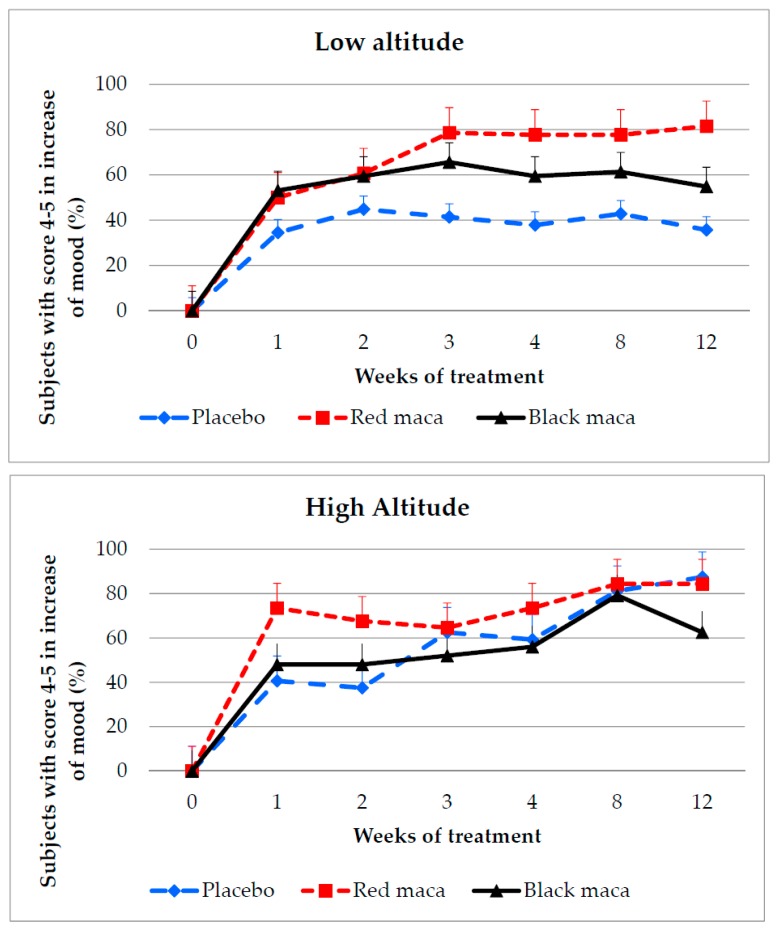
Percentage of subjects perceiving an increase in mood after treatment for 12 weeks with a placebo, spray-dried extract of red maca, and spray-dried extract of black maca. Percentage of subjects with a score for mood of 4–5 (in agreement or completely in agreement that consumption of the product increased their mood) at (**Upper**) low and (**Bottom**) high altitude. LA: *p* = 0.01; 0.01; 0.029; 0.0026 using chi square test at weeks 3, 4, 8, and 12 comparing treatment with placebo, spray-dried extract of red maca, and spray-dried extract of black maca. Placebo: *p* > 0.05 over time (weeks 1 to 12); red maca *p* = 0.0001 over time; black maca *p* > 0.05 over time (weeks 1 to 12). HA: *p* = 0.019; 0.04; and 0.05 using chi square test at weeks 1,2, and 12 comparing treatment with placebo, spray-dried extract of red maca and spray-dried extract of black maca. Placebo: *p* = 0.0001; red maca: *p* = 0.003; black maca: *p* = 0.0002 over time.

At LA, mood increased with both treatments (red maca and black maca), compared with placebo since week 1 (*p* = 0.0017) (Figure 2 upper) and remained elevated at weeks 2, 3, 4, 8, and 12. At week 12, the percentage of subjects with scores of 4–5 for mood was higher with red maca than with black maca (*p* = 0.0002). Treatment with placebo did not affect mood during the 12 weeks of treatment (*p* > 0.05). In contrast, treatment with red maca (*p* = 0.0001) and black maca (*p* = 0.0001) increased the number of subjects with a score of 4–5 in mood. Effect size between placebo and red maca showed a Cohen’s *d* of 1.14 and between placebo and black maca of 0.43, whereas between red and black maca Cohen’s *d* was 0.71.

At HA, treatment with the placebo (*p* = 0.0001), red maca (*p* = 0.003), and black maca (*p* = 0.0002) increased the number of subjects with mood improvement over time. However, differences between the group treated with spray-dried extract of red maca and the placebo were observed at weeks 1, 2, and 4.

Difference between treatment with spray-dried extracts of red maca and black maca was observed as early as week 1 (*p* = 0.0008), with red maca increasing the self-perception of improvement in mood earlier in the study period. At week 12, the percentage of subjects with improved mood was higher after treatment with red maca than with black maca (*p* = 0.0005) but no different with respect to the placebo (Figure 2, bottom). Effect size between placebo and red maca was *d* = 0.68. Between placebo and black maca it was *d* = 0.25 and between red and black maca it was *d* = 0.43.

At weeks 8 and 12 at LA and at HA, red maca increased mood in around 80% of subjects.

#### 2.2.3. Assessment of Energy

**Figure 3:**
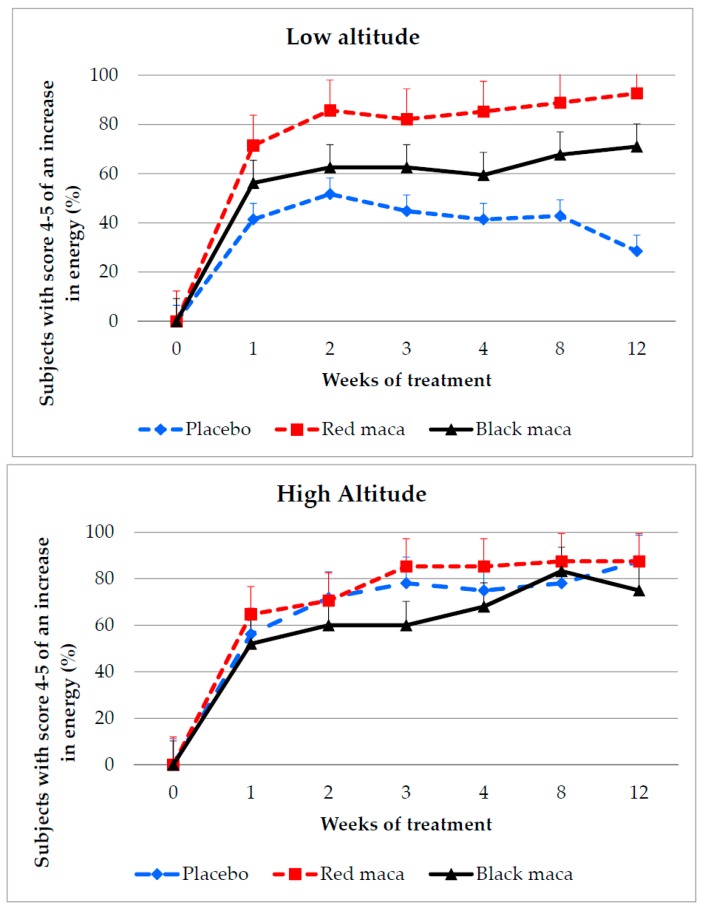
Percentage of subjects perceiving an increase in energy after treatment for 12 weeks with a placebo, red maca, or black maca at low (**Upper**) and high (**Bottom**) altitude. LA: *p* = 0.07; 0.02; 0.01; 0.003; 0.001 and <0.00001 using chi square test at weeks 1, 2, 3, 4, 8, and 12 comparing treatment with placebo, red maca, or black maca. Placebo group: *p* > 0.05 over time (weeks 1 to 12); red maca group: *p* = 0.0001 over time (weeks 1 to 12); black maca group: *p* > 0.05 over time (weeks 1 to 12). HA: *p* > 0.05 between placebo group and groups with red or black maca (chi square test at weeks 1, 2, 3, 4, 8 and 12). Placebo: *p* = 0.0001; red maca: *p* = 0.0001; black maca: *p* = 0.0001 over time (weeks 1 to 12).

At LA, the percentage of subjects that perceived an increase in energy was higher after consumption of spray-dried extracts of black or red maca than with the placebo starting at week 2 and remaining elevated up to week 12 of treatment (Figure 3, upper).

Differences were observed between groups receiving red maca and black maca at weeks 2 (*p* = 0.04), 4 (*p* = 0.029), 8 (*p* = 0.05), and 12 (*p* = 0.036), with better effects observed with red maca. At week 12, more than 90% of the subjects consuming red maca manifested an increase in energy. Cohen’s *d* was 1.90 comparing the group receiving the placebo to that receiving red maca. Treatment with red maca versus the placebo resulted in a Cohen’s *d* of 0.99 and red maca versus black maca resulted in a Cohen’s *d* of 0.55.

At HA, the number of subjects who perceived an improvement in energy increased over time after treatment with placebo, red maca, and black maca (Figure 3, bottom). At week 4, the effect size was higher between the placebo and red maca (*d* = 0.36) than between the placebo and black maca (*d* = −0.19). Between red maca and black maca the Cohen’s *d* was 0.55.

No differences were observed in the responses of the groups with the placebo, red maca, or black maca at each time of treatment (*p* > 0.05). In summary, at HA a placebo effect was observed in the assessment of increase of energy.

#### 2.2.4. HRQL Score

**Figure 4:**
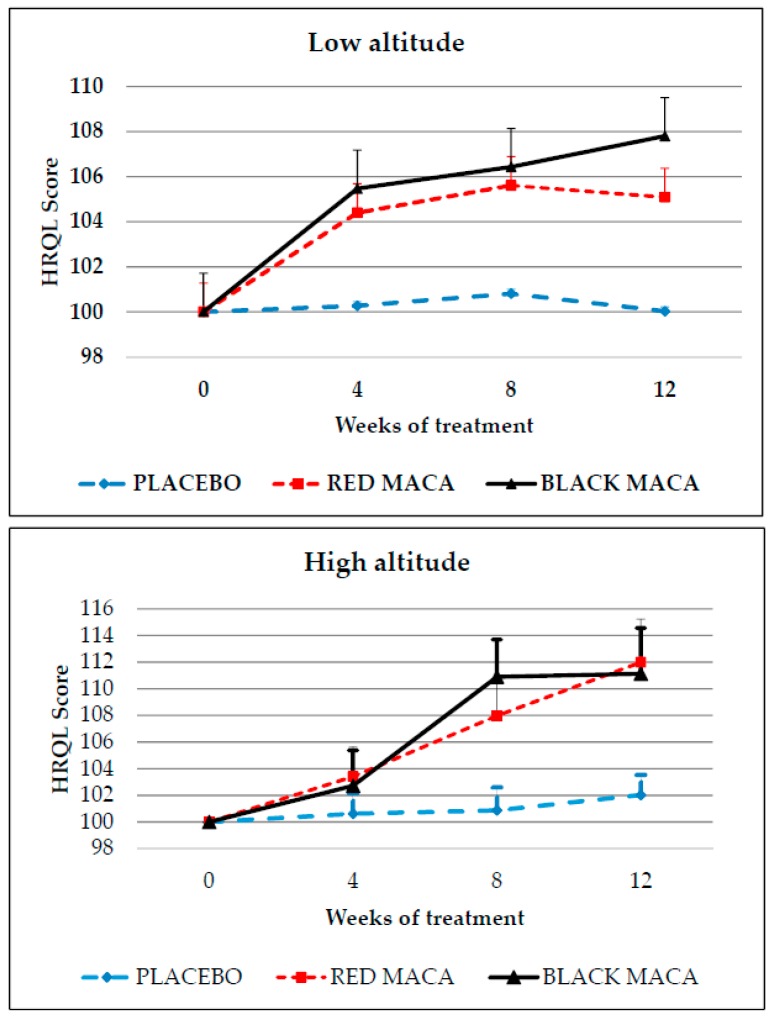
Heath-Related Quality of Life Score (HRQL) in subjects receiving a 12-week treatment of a placebo, spray-dried extract of red maca, or spray-dried extract of black maca at low (**Upper**) or high altitude (**Bottom**). Data are mean ± standard error of the mean. **Upper**: LA: Placebo group: *p* > 0.05 with respect to time 0. Red maca group: *p* < 0.05 at week 8 and *p* < 0.01 at week 12 with respect to time 0 (one tail). Black maca group: *p* < 0.05 at week 4 with respect to time 0 and *p* < 0.01 at weeks 8 and 12 (one tail). *p* < 0.05 comparing red maca group with placebo group at eight and 12 weeks of treatment and *p* < 0.05 at week 4, and *p* < 0.01 at weeks 8 and 12, comparing the group treated with black maca with the group treated with a placebo; **Bottom**: HA: placebo group: *p* > 0.05 with respect to time 0. Red Maca group: *p* < 0.05 at week 8 and *p* < 0.01 at week 12 with respect to time 0. Black maca group: *p* < 0.01 at weeks 8 and 12 with respect to time 0. *p* < 0.05 black and red maca groups at weeks 8 and 12 with respect to the placebo group.

At LA, placebo was unable to modify HRQL score during the 12 weeks of treatment (*p* > 0.05). However, treatment with spray-dried extracts of red maca or black maca increased the HRQL score from the fourth week of treatment onwards with respect to basal values, showing higher values compared with the placebo at weeks 8 and 12 with red maca and at weeks 4, 8, and 12 with black maca (Figure 4 upper). Treatment with black maca and red maca showed a similar response.

At HA, treatment with the placebo did not modify the HRQL score with respect to the basal values. Treatment with red maca increased the HRQL score at the eighth (*p* < 0.05) and 12th (*p* < 0.01) week with respect to basal values. Treatment with black maca increased HRQL score at weeks 8 and 12 with respect to the basal values (*p* < 0.05). Treatment with black maca (*p* < 0.05) and red maca (*p* < 0.05) produced higher increases in HRQL score with respect to the placebo group at eight and 12 weeks (Figure 4 bottom). Participants receiving spray-dried extracts of black maca and red maca showed a similar response.

At LA, higher effect size was observed between groups treated with placebo and black maca (Cohen’s *d* = 0.62; size-effect *r* = 0.199) than those comparing the placebo with black maca (*d* = 0.62; *r* = 0.297). Treatment with black maca and red maca produced a size effect of *d* = 0.208 and *r* = 0.10.

At HA, Cohen’s *d* was higher between the placebo and red maca (*d* = 0.71; size-effect *r* = 0.33) than between the placebo and black maca (*d* = 0.60, and size-effect *r* = 0.28). Effect size was lower between subjects receiving red maca and black maca (*d* = 0.04; size-effect *r* = 0.02).

#### 2.2.5. Pulse Oxygen Saturation (SpO_2_), Body Mass Index (BMI), Glycaemia, Arterial Blood Pressure, and Hemoglobin

Baseline values of SpO_2_ (Data not shown), BMI (data not shown), glycaemia (Table 2), arterial blood pressure (Table 3), and hemoglobin (Table 4) were similar among groups of interventions in Lima as well as in Cerro de Pasco.

Pulse oximetry was assessed prior to maca consumption and at the end of the treatment. Basal SpO_2_ values were higher at LA than at HA (mean ± SD) 98.54 ± 1.04, and 87.92 ± 3.41, respectively (*p* < 0.01). No treatment significantly altered basal SpO_2_ values compared to the placebo in Lima (*p* = 0.817) or in Cerro de Pasco (*p* = 0.374).

Anthropometric measurements were used to obtain body mass index (BMI) and the delta of the final and basal values was used to assess whether maca treatments had an effect on such values. Mean basal BMI was 25.93 ± 1.24 in HA and 25.61 ± 3.95 in LA. The 12-week treatment with spray-dried red maca extract did not have any significant impact on BMI (*p* = 0.56 compared to placebo); neither did the black maca extract (*p* = 0.1 compared to placebo).

At LA, fasting blood glucose levels decreased in the group treated with the placebo at week 12 with respect to basal values (*p* < 0.05), whereas the groups receiving the red maca and black maca treatments had reduced blood glucose level since week 8 that remained lower by week 12 with respect to the basal values. No differences were observed between maca groups and the group treated with the placebo at any time of treatment (*p* > 0.05) (Table 2). At LA, effect size was similar in groups with placebo, red maca, and black maca. At HA, comparing data from week 0 to week 12, the placebo showed a Cohen’s *d* = 0.02 and effect size *r* = 0.01; red maca showed a Cohen’s *d* = 0.40 and an effect size *r* = 0.198 and black maca a Cohen’s *d* = 0.47 and effect size *r* = 0.23.

At HA, the level of glycemia in the group receiving the placebo did not change with time of intervention (*p* > 0.05). The group with red maca reduced glycemia at the 8th week with respect to the basal values (*p* < 0.01). The group treated with black maca had reduced glycemia at the 8th and 12th weeks of treatment (*p* < 0.05). The group treated with black maca had higher glycemia than the placebo group at weeks 0 and 4 of treatment, but at week 12 the difference disappeared (*p* > 0.05) (Table 2).

At LA, SBP did not change over time after treatment with a placebo, black maca, or red maca (*p* > 0.05) (Table 3). SBP in groups treated with black maca or red maca were not different to SBP in the group treated with a placebo at each time of intervention (*p* > 0.05). At HA, administration of a placebo reduced SBP at weeks 8 and 12 (*p* < 0.05). Red maca treatment did not modify SBP during the 12 weeks of treatment. Black maca reduced SBP over time, particularly at weeks 8 and 12 (*p* < 0.01). No differences were observed in the SBP of the groups receiving red maca or black maca with respect to the group receiving the placebo at any time of intervention (*p* > 0.05) (Table 3). At HA, the effect size at week 12 comparing the placebo with black maca showed a Cohen’s *d* = 0.13 and an effect size *r* = 0.06. Comparing the placebo with red maca showed a Cohen’s *d* = 0.25 and an effect size *r* = 0.12. Over time, treatment with a placebo showed a Cohen’s *d* = 0.78 and effect size *r* = 0.36. However, treatment with black maca showed a Cohen’s *d* = 0.91 and effect size *r* = 0.41.

At LA, DBP was unchanged over time after placebo, black maca, or red maca consumption, except at week 4, when DBP was increased in the groups receiving the placebo and red maca (*p* > 0.05) (Table 3). No differences were observed in the groups treated with red maca or black maca with respect to values in the group receiving the placebo (*p* > 0.05).

At HA, DBP increased in the placebo group at week 8 (*p* < 0.05) but not in the group with red maca or black maca (*p* > 0.05). No differences were observed in the groups treated with red maca or black maca with respect to values in the placebo group (*p* > 0.05) (Table 3).

At LA, hemoglobin values remained unchanged in all groups at all times (*p* > 0.05) (Table 4). At HA, treatment with a placebo did not change the levels of hemoglobin (*p* > 0.05). However, treatment with red or black maca decreased hemoglobin starting at the 4th week and maintained this lower value without further modification over time. Hemoglobin value was lower than the placebo only in the group receiving black maca at the 4th, 8th and 12th week of treatment (Table 4). At HA, the effect size at week 12 comparing the placebo with black maca showed a Cohen’s *d* = 0.77 and an effect size *r* = 0.36. Comparing the placebo with red maca showed a Cohen’s *d* = 0.25 and an effect size *r* = 0.12.

#### 2.2.6. Chronic Mountain Sickness Score

**Figure 5:**
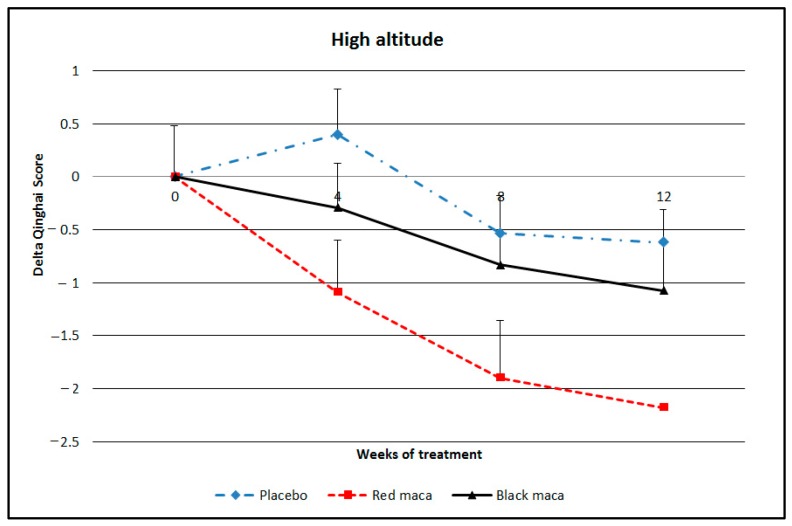
Delta of Qinghai score for Chronic Mountain Sickness (CMS) diagnosis in adult human subjects after a 12-week treatment with spray-dried extract of maca (red or black) or with a placebo at high altitude. Data are mean ± standard error of the mean. Placebo: *p* > 0.05 over time. Red maca: *p* < 0.05 at week 4 and *p* < 0.01 at weeks 8 and 12 with respect to values at time 0. Black maca: *p* < 0.05 at weeks 8 and 12 with respect to values at time 0. Red maca group: *p* < 0.05 at weeks 4 and 8 with respect to the placebo and *p* < 0.01 with respect to the placebo at week 12.

Treatment with placebo did not modify the Qinghai score during the 12 weeks of treatment (*p* > 0.05). Red maca reduced the Qinghai score at weeks 4 (*p* < 0.05), 8 (*p* < 0.01), and 12 (*p* < 0.01) of treatment. Black maca reduced the Qinghai score at weeks 8 and 12 (*p* < 0.05) (Figure 5). Compared with the placebo, treatment with spray-dried extract of red maca significantly reduced the Qinghai score since the 4th week. Results with spray-dried extract of black maca were not different from the placebo (*p* > 0.05). Effect size at HA comparing treatment with placebo and black maca showed a Cohen’s *d* = 0.61 and effect size *r* = 0.29. Between placebo and black maca there was a Cohen’s *d* = 0.19 and effect size *r* = 0.09. Finally, between treatment with red maca and black maca there was a Cohen’s *d* = −0.36 and effect size *r* = −0.18.

### 2.3. Secondary Outcomes

#### 2.3.1. Acceptability and Adverse Effects

Acceptability was similar between groups consuming spray-dried extracts of maca or placebo at low altitude (*p* > 0.05) and at high altitude (*p* < 0.05). However, acceptability was different between both places (*p* < 0.05). At LA, acceptability was observed in less than 80% of the studied population but without differences between subjects consuming the placebo or spray-dried extracts of red or black maca. At HA, more than 80% of the population liked the products consumed. No differences were observed between placebo group and groups consuming spray-dried extracts of red or black maca (*p* > 0.05).

Metabolite levels in the maca samples are the relative intensities of the characteristic peaks of the compounds to the internal reference TSP; the proton number contributing to the peak is not taken into consideration. Clear differences in composition between spray-dried extracts of black maca and red maca are observed for sucrose, macamides, GABA, and fatty acids.

Since the first week of consumption, the groups consuming spray-dried extracts of black and red maca showed the highest acceptability. Dislike of the taste of the products (scores −1 to −4) was observed in percentages ranging from 0%–4%.

No serious adverse effect was reported in the groups treated with a placebo, red maca, or black maca at LA and at HA. To the statement, “I am healthy without any systemic or functional impairment,” of the low-altitude subjects who disagreed or completely disagreed (scores 1 and 2), 0%–3.6% were in the placebo group, 0%–3.7% received the spray-dried extract of red maca, and 0%–3.2% received black maca; of the high altitude subjects with scores of 1 or 2, 0%–15.6% were in the placebo group, 0%–14.7% received spray-dried extract of red maca, and 0–8% received spray-dried extract of black maca.

#### 2.3.2. Effect of Altitude

No difference between the placebo and spray-dried extracts of red or black maca was observed at LA and at HA (*p* > 0.05). The percentage of subjects scoring 1 and 2 did not change over time at LA (*p* < 0.05) but decreased over time in the groups treated with the placebo (*p* = 0.00001), red maca (*p* = 0.00003), and black maca (*p* = 0.0059) at HA.

#### 2.3.3. Blinding

Analysis of participants’ response to the question of whether they thought they had received maca or the placebo indicated that participants were blinded to study assignment. Participants receiving the placebo, red maca, or black maca believed they had received the placebo, maca, or reported not knowing what treatment they had received in a similar proportion. There was no difference in blinding between the intervention groups (Chi square test = 4, 62; *p* = 0.327).

## 3. Discussion

This study was primarily designed to determine the efficacy of oral consumption of extracts of black maca or red maca, a cruciferous plant, during a 12-week period in a double-blind placebo-controlled trial at LA and HA. Secondary objectives of the study were the determination of acceptability, adverse effects, and effect of altitude on outcomes after maca intervention.

### 3.1. Primary Outcomes: Efficacy

During the period of the study, consumption of spray-dried extracts of black and red maca increased self-perception of sexual desire, mood, and energy. This is in accordance with experimental studies and results in human beings with unidentified maca [8].

This trial was also able to demonstrate that spray-dried extracts of black maca and red maca have different biological properties. In fact, self-perception of increase of sexual desire, mood, and energy, and reduction in CMS score were more relevant with red maca than with black maca. This is in accordance with different properties shown for black maca and red maca in experimental designs [8]. Black maca demonstrated better response with hemoglobin levels at high altitude. Both maca phenotypes have similar responses in HRQL score.

Although red maca increased sexual desire more than black maca, the effect seems to be modest since it represented only 50% of the subjects. Moreover, a placebo effect was observed particularly at HA. In a previous study using gelatinized maca in men at LA, sexual desire increased only in 42% and after eight weeks of treatment [16]. Maca dry extract supplementation was also useful to improve subjective perception of general and sexual well-being in adult men with mild erectile dysfunction [17]. Another study using maca flour at a dose of 3.3 g/day for six weeks was unable to show an increase in sexual desire in post-menopausal women [15]. Stone et al. [14] showed an increase in sexual desire at week 4 using an extract of unidentified maca. Although extract of maca, particularly the red phenotype, seems to be better than flour or gelatinized maca, the results are still modest.

Maca shows greater effects on mood and energy. In fact, over 80% of the population reported increased mood and energy following maca consumption.

Among the most notorious compounds in maca are macamides, a group of fatty acid lipids. These compounds seem to act on a widely expressed signaling system known as the endocannabinoid system (ECB) [18,19]. Macamides inhibit anandamide degradation through the inhibition of fatty acid amide hydrolase (FAAH). Anandamide acts on the CB1 receptor. Maca’s effects on mood might be linked to its role on the CB1 receptor since other FAAH inhibitors have been shown to act as antidepressants by enhancing central serotonergic and noradrenergic transmission and promoting neurogenesis in the hippocampus [20].

The presence of GABA might also explain the elevated mood [21] after the 12 weeks of treatment. GABA was higher in red than in black maca and this may explain the differences in the response on mood. Indeed, the present study showed that treatment with red maca was more effective at increasing mood than black maca.

Choline is another compound present in spray-dried extracts of maca. Choline supplementation improves neurocognitive functioning [22,23,24]. Choline also plays a critical role in hepatic function and systemic lipid metabolism [25]. Mood is also improved after choline supplementation [26].

Choline acts in the brain as an agonist of the alpha 7 nicotinic acetylcholine receptor (α7 nAChR) [27]. Choline is also antinociceptive [27] and this property may improve the score in the HRQL questionnaire. The generic health-related quality of life (HRQL) questionnaire used for assessment health status [12] showed that the score increased with treatment with spray-dried extract of black or red maca compared to the placebo. This was an effect of maca and not a placebo effect. This finding confirms previous findings that populations consuming maca at HA have better HRQL scores than populations not consuming maca at the same altitude [8,12].

Our finding of increased HRQL scores after maca consumption is also in concordance with the absence of serious or severe adverse effects due to consumption of spray-dried extracts of red or black maca reported in the present study.

In the present study, at LA or at HA, systolic or diastolic blood pressure did not change after consumption of spray-dried extracts of red or black maca compared to the placebo. In a previous study in postmenopausal Hong Kong Chinese women, there was a significant reduction in diastolic blood pressure over 12 weeks of treatment with 3.3 g/day of maca [15]. However, there is a report in patients with metabolic syndrome indicating that maca administration at a dose of 0.6 g/day for 90 days resulted in a moderate elevation of AST and diastolic blood pressure [28]. There is no other study confirming this finding. In fact, in a previous study, no increase in arterial blood pressure occurred in healthy men who took gelatinized maca at doses of 1.5 of 3.0 g/day for 90 days [1]. The fact that maca contains high amounts of potassium and low of sodium [1] and inhibitory activity of ACE [7] suggests that it is feasible that maca may reduce arterial blood pressure rather than increase it.

Spray-dried extract of black maca reduced fasting glucose levels over time whereas in the placebo group they did not change during the treatment period of 12 weeks at LA and at weeks 4 and 8 at HA. This result corroborates previous results from experimental studies in which black maca reduced fasting blood glucose levels in mice with streptozotocin-induced diabetes [5]. In addition, maca, as well as rosiglitazone, significantly improved glucose tolerance, decreased the AUC (area under the curve) of glucose, and reduced glycemia [29]. In our study, black maca reduced glycemia at both LA and HA. A placebo effect also occurred for glycemia reduction over time: glycemia was significantly lower at HA than at LA, confirming results previously reported [30].

Hemoglobin was another primary outcome assessed, since scientific publications without scientific evidence suggest maca has been used for centuries in the Andes to treat anemia [15]. This assumption is probably due to the presence of iron in maca [1]. However, our results at LA show that 12 weeks of treatment with spray-dried extract of black or red maca has no improving effect on hemoglobin level, suggesting that maca is not useful in treating anemia at LA.

On the contrary, our results indicating a reduction in hemoglobin levels at HA after black maca consumption are remarkable since high hemoglobin levels at HA are associated with chronic mountain sickness, a condition of lack of adaptation to HA compromising almost 20% of the population living over 4000 m [11]. Previously, a cross-sectional study in a population close to Cerro de Pasco (over 4000 m) showed that maca consumers have lower CMS scores than those non-maca consumers living in the same place [8]. Data suggest that red maca could be acting on signs and symptoms whereas black maca is acting on high hemoglobin levels. Our results suggest that this is an effect based on maca. The consumption of maca, particularly the red phenotype, could be an important alternative in cases of CMS.

Our study shows a placebo effect in several variables studied, although these were not consistent over time. This placebo effect was also observed in other studies with maca [15,31], but our results are consistent, in which several of the primary outcomes studied were different with maca than with the placebo.

Our results are in accordance with other authors who suggest that the placebo effect should be part of alternative medicine [32].

Recent findings showed that neurotransmitter pathways might mediate placebo effects [33]. The higher placebo effect observed at HA than at LA is remarkable. Genetic signatures have been identified in those subjects responding to placebo [34] and this may explain the differences between populations at LA and HA. At HA, placebo administration to headache sufferers inhibited the nocebo-related component of pain and prostaglandin synthesis [35]. This may explain the reduction of CMS score with placebo in headache as part of the symptoms included in the CMS test.

### 3.2. Secondary Outcomes: Acceptability

Most of the subjects included in the present study were satisfied with the taste of the product consumed independently if they consumed a placebo or a spray-dried extract of maca. This high satisfaction occurred as early as the first week of treatment and reached its highest values at weeks 8 and 12.

This is an important finding since cruciferous vegetables are bitter, which might be off-putting to consumers [36]. Thus, high acceptability of consuming a spray-dried extract of maca, another cruciferous vegetable, might be important to promote its consumption as a value-added product. Perceived bitterness of cruciferous vegetables varies from person to person, which suggests a genetic contribution in acceptability [37]. Glucosinolates and metabolites, compounds present in maca (Table 1), impart the characteristic bitter taste and pungent odor of cruciferous vegetables [38,39]. Maca flour has a pungent smell and bitter taste and may result in aversion in persons not accustomed to its consumption.

Our results showed differences in acceptability between populations at LA and at HA. It is possible to think that subjects at LA are not as accustomed to the maca flavor as those living at HA. Our study used spray-dried extracts of black maca or red maca instead of flour, resulting in an improvement of the taste with maintenance of biological activity.

### 3.3. Adverse Effects

It is common to find severe adverse effects with medicinal plants’ usage [40]. For such reason, since interest in maca, a nutraceutical plant, has increased worldwide, it is important to know its adverse effects, particularly in populations with little or no experience with its consumption.

Another secondary outcome of this study was to determine the adverse effects after consumption of spray-dried extracts of maca and compare data with those obtained after placebo consumption during an intervention period of 12 weeks. There were no severe or serious adverse effects due to consumption of an extract of red or black maca, and no subject discontinued the trial due to an adverse effect. The discontinuation rate was similar in the three groups of treatment at LA and at HA. Most occurred at the first month due to reasons besides adverse effects or disagreement with the products. Moreover, the percentage of people missing the follow-up was similar between groups consuming the placebo, black maca extract, or red maca extract.

Previous findings in experimental animals receiving different phenotypes of maca (black, red, or yellow) showed no acute toxicity at ≤17 g of dried hypocotyls/kg body weight (BW) [1]. Similarly, rats treated chronically for 84 days with 1 g maca/Kg BW showed no adverse side effects and a histological picture of the liver similar to that observed in controls [41]. Gelatinized maca given for 12 weeks to healthy adult men and women also showed that consumption was safe [13]. All data taken together suggest that in humans the consumption of spray-dried extract of red or black maca at the dose used in the present study seems to be safe. This dose is used in Peru in self-care and/or medical practice.

The use of 3 g of the spray-dried extract was calculated from data obtained in experimental animals using the body surface normalization method [42].

The present study provides data that could be the basis for the use of extracts of black or red maca (*Lepidium meyenii*) in adult human subjects in possible clinical therapies.

## 4. Materials and Methods

### 4.1. Subjects and Settings

The study includes men and women from Cerro de Pasco, located 4340 m above sea level (m.a.s.l) in the Peruvian Central Andes, and those from Lima (150 m.a.s.l) on the Peruvian Central Coast. All subjects eligible were included in the analysis as far as their enrollment lasted (analysis by intention-to-treat). Figure 1 (A: Lima was considered as low altitude, LA; B: Cerro de Pasco was considered as high altitude, HA) shows the details of recruitment and the number of people who missed the follow-up.

Eligible subjects were not receiving medication or consuming maca during the last three months and were residents for at least 10 years in the place of the study (Lima or Cerro de Pasco). Pregnant women, chronic smokers, women with amenorrhea not related to pregnancy or menopause, and subjects with dyslipidemia, diabetes mellitus, metabolic syndrome, hypertension, or with a low health status score (<1000 points on the HRQL questionnaire) were excluded from the study.

All subjects gave their informed consent for inclusion before they participated in the study. The study was conducted in accordance with the Declaration of Helsinki, and the protocol was approved by the Ethics Committee of the Universidad Peruana Cayetano Heredia and the National Institute of Health (NIH) belonging to the Peruvian Ministry of Health (Project identification code 61697).

### 4.2. Recruitment, Randomization, Concealment Allocation, and Lost to Follow-Up

Recruitment of subjects occurred through local advertisement in universities, offices, and mass media in Lima and Cerro de Pasco. The study assessed for eligibility 145 adults at LA and 126 at HA (*n* = 271). From these we recruited 197 volunteers aged 18–65 years; they were randomized equally to obtain 96 subjects (32 subjects per group) at low altitude and 105 subjects (35 subjects per group) at high altitude. For the study was used stratified randomization controlling age (≤40 years and >40 years). The sequence of assignments was concealed until interventions were assigned. Then, using simple randomization within each block, subjects were assigned to one of the three groups of interventions. One investigator generated the randomization list in Lima and another in Cerro de Pasco. Two of the randomized subjects from LA and six from HA did not start the study for personal reasons. The final randomized sample included 94 subjects at LA and 99 subjects at HA. The age of subjects in each group of intervention was similar at LA and at HA. The proportion of men and women was similar among groups of interventions at LA (Chi square test = 0.74; *p* = 0.68) and at HA (Chi square test = 0.40; *p* = 0.81).

Allocation concealment: Products were placed in a similar container (sachet) with 3 g each. The sachet was not labeled. Thirty sachets were deposited in one display. All displays were similar in size and of white color. The display was labeled with A, B, or C.

For concealment, the manufacturer put on a piece of paper the kind of product designated by each label and sealed the information in an envelope. This was kept closed up to the end of the study and analysis. No participants, investigators, or staff members who were collaborating with the study knew the meaning of each label. Each participant received only the sachets belonging to his/her group. They did not know if they belonged to group A, B, or C.

**Figure 6:**
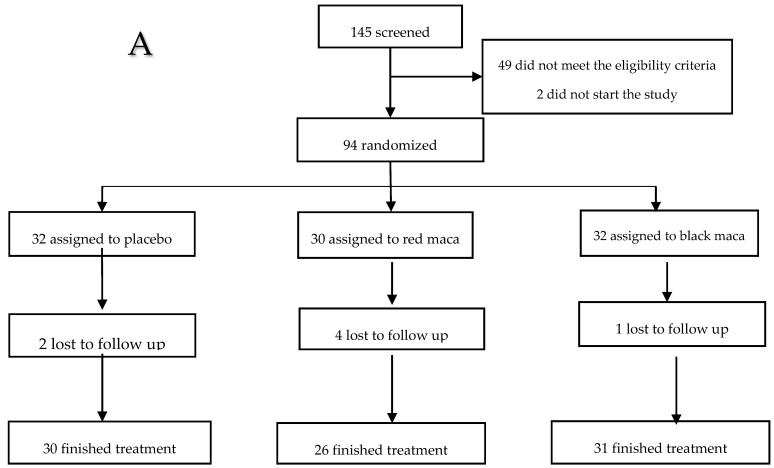

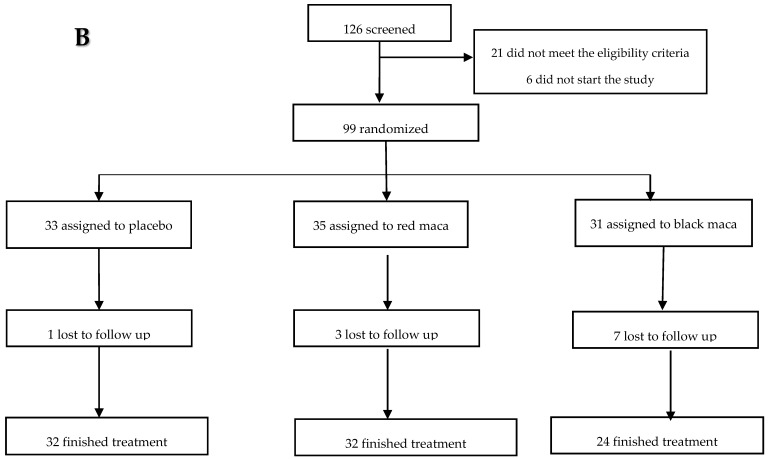
Flow diagram following the recruitment, enrollment, and number of subjects that finished treatment of the clinical trial at low altitude (**A**) and high altitude (**B**). Low altitude: Chi square: 0.11; *p* = 0.99 with respect to subjects who failed to complete the follow-up among different groups of treatment. High altitude: Chi square: 0.6; *p* = 0.99 with respect to subjects who failed to complete the follow-up among different treatment groups.

At LA, seven randomized and enrolled subjects withdrew from the study between the 4th and 8th week. Two belonged to the placebo group, four to the red maca group, and one to the black maca group (*p* > 0.05). At HA, 11 subjects withdrew from the study between the 4th and 8th week. One belonged to the placebo group, three to the red maca group, and seven to the black maca group (*p* > 0.05) (Figure 6a,b).

Eighty-seven subjects finished treatment at LA and 88 at HA. Subjects left the study mainly due to difficulties of attending the day of sampling due to work. Other subjects left the study due to travel to another place for different reasons. The number lost to follow-up were similar among the different treatment groups at LA (*p* = 0.99) and at HA (*p* = 0.99) (Figure 6). According to the Food and Drug Administration (FDA), the total number of subjects and patients included in Phase 1 studies varies with the drug, but is generally in the range of 20 to 80 [43]. In the present study, all groups met these criteria.

After interventions, participants were asked to indicate if they thought they had received the placebo or maca. This was performed for assessment of quality of blinding in participants.

### 4.3. Design

This was a 12-week randomized, double-blind, placebo-controlled study comparing the efficacy, acceptability, and safety of oral administration of spray-dried extracts of black maca, red maca, or a placebo in adult human subjects living at 150 m (Lima, Peru) or 4340 m altitude (Cerro de Pasco, Peru). In each place, around one-third of the subjects received spray-dried extracts of black maca, red maca, or a placebo. Men and women were randomly assigned to each group.

All subjects received information on the purpose and procedures of the study. The participation of the volunteers included, among other things, filling out of a questionnaire and allowing sampling of venous blood. In Cerro de Pasco, 8% declared that they were smokers but less than one pack a year. In Lima, 16.8% smoked (41% smoked one packet a year, 35% one packet a month, and 24% less than one packet a month).

A CONSORT Herbal Extension checklist for the reporting of this study is inserted as a supplementary file.

### 4.4. Intervention

This study used both spray-dried maca extracts or a placebo placed in individual sealed sachets, in an amount of 3 g. Spray-dried extracts of maca or placebo have the same appearance. Each subject only received one kind of product. Subjects were unable to differentiate between the products. Oral administration of sachets of maca extracts or the placebo occurred daily for a period of 12 weeks. The dose of 3 g per subject was calculated by extrapolating from data from previous animal studies [3,4,5,6].

Follow-up occurred each week during the first four weeks and then at week 8 and 12. On each visit we recorded information about incidents. Adherence was monitored through sachets delivered each visit. Each subject returned empty sachets and they received new, full sachets.

The subjects were asked to consume the same diet they normally eat throughout the period of the study. However, they were told not to consume additional maca products.

Practitioners in charge of the intervention have all approved the CITI training course for research in human subjects. All have experience in clinical trials with medicinal plants, ranging from one to 15 years. To enhance the quality of observations, staff members were trained prior to interventions.

### 4.5. Products

The plant was authenticated by Camilo Díaz, Botanist, Section of Pharmaceutical Sciences, the Faculty of Sciences and Philosophy, Universidad Peruana Cayetano Heredia. The voucher specimen (HEPLAME-MG-2015) was placed at the Section of Pharmaceutical Sciences, University Peruana Cayetano Heredia. Authentication was done by morphological characteristics. In addition, authentication was performed by the presence of glucosinolates and macamides in the hypocotyls of maca by nuclear magnetic resonance (NMR). For this research, spray-dried hydro alcoholic extracts of black maca or red maca was used. For extract preparation, 70% ethanol was used. Maltodextrin was used as the placebo. The dried hypocotyls of maca were used to prepare the extracts. Twenty grams of dried hypocotyls of maca represents almost three grams of the spray-dried extract of maca. Enterprise A-1 del Perú, Industrial Comercial SAC was in charge of preparation of the maca extracts and placebo. The excipient for maca extract was maltodextrin. For this reason, maltodextrin was used as the placebo. According to the protocol, the sachets containing 3 g of powder each were packed in boxes containing 30 sachets by Artpack Perú SAC. Each box (display) was labeled as A, B, or C. Boxes had been kept under appropriate storage conditions at the Research and Development Laboratory building at the Universidad Peruana Cayetano Heredia in Lima and at the Laboratory of the High Altitude Research Institute in Cerro de Pasco. For standardization, maca extracts were submitted to bioassays in mice and rats to determine functional measures of activity.

Staff taking blood samples only knew that volunteers received treatment A, B, or C, but not which kind of compound they were receiving.

Metabolites in spray-dried extracts of black and red maca were screened using nuclear magnetic resonance (NMR) and chemometric analysis as previously reported [44]. In total 16 metabolites were identified in the methanolic extract of maca, i.e., 1. adenine; 2. alanine; 3. choline; 4. fatty acids; 5. formic acid; 6. fumaric acid; 7. glutamine; 8. glucotropaeolin; 9. macamides; 10. malic acid; 11. proline; 12. sucrose; 13. uridine; 14. valine; 15. phytosterols; and 16. γ-aminobutyric acid (GABA).

### 4.6. Primary Outcomes: Efficacy

#### 4.6.1. Assessment of Sexual Desire, Mood, and Body Energy Self-Report

A Likert test was used for self-perception assessment of increase in sexual desire, mood, and body energy as an effect of treatment. Participants were asked at the end of each week during the first four weeks and at the end of the 8th and 12th week to qualify the effect of treatment on their sexual desire, mood, and body energy in three 5-point Likert scales ranging from “disagree strongly” (score 1) to “agree strongly” (score 5). For analysis, subjects answering with a score of four or five were considered to be in agreement with an increase in sexual desire, mood, or body energy.

#### 4.6.2. Health Status Score

A generic health-related quality of life (HRQL) questionnaire was used for evaluation of self-perception about health status (SF-20). The health questionnaire based on 20 questions or items about health included seven dimensions: physical functioning scale, role physical scale, bodily pain scale, general health, vitality scale, role emotional scale, and mental health scale. This SF-20 questionnaire is based on a Spanish version of a validated previous HRQL questionnaire (SF-36) that contains 36 questions [45]. All items are scored on a scale of 0 to 100, in which higher scores indicates better HRQL.

The SF-20 questionnaire has five items related to general health, five items related to physical activities associated with current health status, two items related to limitations on work or other regular daily activities as a consequence of reduced physical health, two items on bodily pain, one item about vitality, and five items on mental health [12]. Previously, this questionnaire was validated in populations living at low and high altitudes in Peru [12]. Each item has a maximal value of 100; the maximum value for the overall test is 2000 points. The same questionnaire was used in a cross-sectional study on the effect of maca consumption in a population living in the Peruvian Central Andes [12]. For analysis, basal values are referred to as 100% and values at 4, 8, and 12 weeks are related to this basal value.

#### 4.6.3. Biological Measurements

Fasting blood glucose, arterial blood pressure, hemoglobin, and pulse oxygen saturation (pSO_2_) were also measured to assess the efficacy of the consumption of spray-dried extracts of red or black maca compared to placebo administration. These measurements occurred before the trial and at weeks 4, 8, and 12 of the maca extracts or placebo consumption.

For glucose measurement, a portable glucometer Accu-Chek (ROCHE, Indianapolis, IN, USA) was used. This method is based on the activity of the glucose oxidase enzyme. Data are measured in mg/dL.

Hemoglobin value (g/dL) was obtained in situ using the HemoCue system (Anglholm, Sweden).

The pulse oxygen saturation (%) was measured in the second left finger of each volunteer using a Nellcor N-20 pulse oxymeter (Pleasanton, CA, USA).

Sitting arterial blood pressure (BP) (mm Hg) was measured in the left arm using an aneroid sphygmomanometer. Systolic (SBP) and diastolic (DBP) blood pressure were obtained from each subject at the beginning of the trial and on each visit to the laboratory.

#### 4.6.4. Anthropometric Measurements

Anthropometric measurements such as body weight and height were performed on each volunteer. Body weight (Kg) was measured while the participants were wearing light clothing and no shoes with a TANITA Body Composition Analyzer BF 350 (Tanita Corporation, Tokyo, Japan). Height was determined with a stadiometer to the nearest 0.1 cm. With these data, body mass index (BMI) was calculated in kg/m^2^.

Weights of the subjects were at the same time of day at the start of the study and after four, eight, and 12 weeks of interventions.

#### 4.6.5. Chronic Mountain Sickness (CMS) Score

All volunteers completed at each visit a test of seven signs and symptoms of chronic mountain sickness (CMS). The signs/symptoms refer to problems associated with life at high altitude: (1) breathlessness and/or palpitations; (2) sleep disturbance; (3) presence of cyanosis; (4) venous dilatation; (5) paresthesia; (6) headaches; and (7) tinnitus. A value of zero (0) was assigned as absence of the sign or symptom and values from 1, 2, and 3 to positive answers (mild, moderate, and severe magnitude, respectively).

The score of clinical signs and symptoms (CMS clinical score) was defined according to the sum of points obtained for each of the seven signs/symptoms of CMS. For diagnosis of chronic mountain sickness, to the clinical score was added a value of 3 if hemoglobin value was ≥21 g/dL in a man or ≥19 g/dL in a woman and the total score, including the score for Hb value, was considered the “Qinghai CMS Score” [46].

### 4.7. Secondary Outcomes: Acceptability and Adverse Effects

#### 4.7.1. Acceptability

Participants were asked each week during the first four weeks and at the end of the 8th and 12th week to score the taste of the product consumed on a nine-point scale ranging from “extremely unpleasant” to “extremely pleasant.” This scale is reliable for assessment of taste preference for oral nutrition supplements [47]. Acceptability is scored as 1–4, whereas disagreement was scored as −1–−4. Neither like nor dislike is scored as zero. Acceptability was also assessed if it was different at low altitude and high altitude.

#### 4.7.2. Safety Self-Recording

Subjects were asked about their experience of adverse side effects. For safety assessment, adverse events were documented at all visits using a Likert test. The scale ranges from 1 to 4 (completely disagree to completely agree) in response to the statement “I am healthy, without any systemic or functional affection.” In addition, each subject was asked on each visit about any adverse effects.

### 4.8. Statistical Analysis

Data were analyzed using the Stata 10 version software (Stata Corp LP, College Station, TX, USA).

Continuous variables are shown as means ± standard error of the mean and categorical variables as absolute frequencies. The percentage of subjects who reported increased sexual desire, mood, and energy due to intervention was calculated. Standard error was also calculated for each result. The chi-square test was used to evaluate the associations between the categorical variables and the Student’s *t* test and ANOVA were applied to evaluate the differences in mean values of normally distributed data between two means or more than two means, respectively. Furthermore, to compare differences between the two means after ANOVA, post hoc analyses were performed. For the assessment of acceptability, we used data from scores 1 to 4; for adverse effects we used data from scores 1–2; for the effect on sexual desire, mood, and energy we used data from scores 4–5.

We measured the effect size by using the Cohen´s *d* statistics and effect size r to determine the magnitude of treatment on primary outcomes. Effect size is defined as “small” when *d* = 0.2, “medium” when *d* = 0.5, and “large” when *d* = 0.8 [48]. Effect size was calculated when the data show statistical significance with respect to the placebo and/or change over time (from time zero to week 12.

The level of significance was defined as *p* < 0.05.

## 5. Conclusions

The present study has demonstrated that consumption of a spray-dried extract of red and black maca for 12 weeks resulted in an improvement of mood, energy, and health status, and a reduced CMS score.

Fatty acids and macamides were higher in spray-dried extracts of black maca than in red maca. GABA predominated in spray-dried extracts of red maca. Effects on mood, energy, and CMS score were better with the spray-dried extract of red maca. Maca reduced hemoglobin levels only in high altitude, where abnormally high hemoglobin values are related to HA maladaptation. No effect on Hb levels was observed in LA. In addition, consumption of spray-dried extracts of red and black maca is safe and both have good acceptability, but better at high altitude.

## Figures and Tables

**Table 1:** Composition of spray-dried extracts of red and black maca screened using nuclear magnetic resonance.

Compound	Maca Samples	
	Black Maca	Red Maca
Formic acid	4.10	6.94
Adenine	6.23	5.06
Fumaric acid	13.79	15.43
Uridine	10.82	10.71
Sucrose	1632.36	1368.35
Glucotropaeolin	77.50	72.08
Macamides	55.21	39.86
Choline	327.44	366.99
GABA	40.09	76.59
Malic acid	83.88	92.81
Glutamine	132.01	118.27
Proline	875.25	832.98
Fatty acids	504.55	378.36
Alanine	156.22	152.41
Valine	111.48	107.61
Phytosterols	24.23	21.10

**Table 2:** Fasting glycemia (mg/dL) in adult human subjects who consumed spray-dried extracts of maca (red or black) or a placebo at low (LA) and high altitude (HA).

Time of Consumption (Weeks)	Placebo		Red Maca		Black Maca	
	LA (32)	HA (33)	LA (30)	HA (35)	LA (32)	HA (31)
0	90.78 ± 1.38	71.48 ± 2.25	92.81 ± 1.48	75.00 ± 2.47	91.61 ± 1.12	78.96 ± 2.50 ^a^
4	89.77 ± 1.40	69.84 ± 1.70	90.33 ± 1.34	72.66 ± 2.04	90.23 ± 1.26	74.66 ± 1.60 ^b^
8	88.90 ± 1.42	67.59 ± 2.11	87.29 ± 1.60 **	67.77 ± 2.04 **	86.71 ± 1.31 **	70.59 ± 2.44 **
12	84.87 ± 1.49 **	71.12 ± 2.48	86.15 ± 2.24 **	69.62 ± 2.09	86.03 ± 1.77 **	72.33 ± 2.86 **

Data are mean ± standard error of the mean. ** *p* < 0.05 with respect to values at time 0. ^a^ *p* < 0.01; ^b^ *p* < 0.05 between black maca group and placebo group. In parentheses is the number of subjects starting the study per group of treatment and place of the study.

**Table 3:** Systolic and diastolic arterial pressure of subjects who consumed maca or a placebo at low and high altitude.

Treatment	Time 0				4th Week				8th Week				12th Week			
	SBP		DBP		SBP		DBP		SBP		DBP		SBP		DBP	
	LA	HA	LA	HA	LA	HA	LA	HA	LA	HA	LA	HA	LA	HA	LA	HA
Placebo	101.6 ± 2.33	101.75 ± 2.14	66.56 ± 1.86	62.96 ± 2.15	102.9 ± 1.62	100.18 ± 1.83	71.94 ± 1.57	63.96 ± 2.10	99.67 ± 1.73	96.43 ± 1.48 **	68.67 ± 1.47	69.18 ± 1.67 **	100.33 ± 1.86	94.87 ± 1.95 **	65.50 ± 1.77	68.12 ± 1.59
Red maca	97.78 ± 1.82	103.71 ± 2.12	66.30 ± 2.00	64.91 ± 1.83	103.33 ± 1.41	99.58 ± 1.49	71.85 ± 1.58	65.12 ± 1.94	102.86 ± 1.61	98.15 ± 2.34	70.36 ± 1.66	67.34 ± 2.01	101.54 ± 1.41	98.43 ± 2.46	69.62 ± 1.48	68.12 ± 1.81
Black maca	98.71 ± 1.92	103.62 ± 1.96	67.90 ± 2.30	63.28 ± 2.45	100.97 ± 1.79	99.96 ± 1.46	70.00 ± 1.77	63.75 ± 1.17	103.87 ± 1.63	95.08 ± 2.22 *	70.16 ± 1.75	68.33 ± 1.94	101.33 ± 1.59	93.33 ± 2.38 *	69.33 ± 1.22	64.16 ± 1.69

SBP: Systolic blood pressure (mm Hg); DBP: Diastolic blood pressure (mm Hg); LA: low altitude; HA: high altitude. Time 0: Basal values. Data are mean ± standard error of the mean. * *p* < 0.01; ** *p* < 0.05 with respect to basal values (Time 0). *p* > 0.05 between maca group and placebo group.

**Table 4:** Hemoglobin concentration (g/dL) in adult human subjects who consumed maca or a placebo at low altitude (LA) and high altitude (HA).

Time of Consumption (Weeks)	Placebo		Red Maca		Black Maca	
	^¶,¥^ LA	HA ^¥^	^¶,¥^ LA	HA	^¶,¥^ LA	HA
0	13.97 ± 0.20	17.80 ± 0.36	13.58 ± 0.24	18.21 ± 0.38	13.40 ± 0.23	17.47 ± 0.43
4	13.83 ± 0.28	18.03 ± 0.30	13.69 ± 0.27	17.28 ± 0.36 *	13.61 ± 0.21	16.40 ± 0.38 *^,a^
8	13.68 ± 0.28	17.96 ± 0.29	13.64 ± 0.27	17.10 ± 0.37 *	13.48 ± 0.23	16.23 ± 0.38 **^,a^
12	14.48 ± 0.28	17.91 ± 0.35	14.09 ± 0.28	17.38 ± 0.38 **	13.73 ± 0.23	16.22 ± 0.49 *^,a^

Data are mean ± standard error of the mean. ^¶^ *p* > 0.05 between maca groups and placebo group. ^¥^ *p* > 0.05 with respect to time of treatment. * *p* < 0.01; ** *p* < 0.05 with respect to values at time 0. ^a^ *p* < 0.01 between the group receiving black maca and the group receiving a placebo.
